# Reduced System Complexity of Heart Rate Dynamics in Patients with Hyperthyroidism: A Multiscale Entropy Analysis

**DOI:** 10.3390/e24020258

**Published:** 2022-02-10

**Authors:** Jin-Long Chen, Hsuan-Shu Shen, Shih-Yi Peng, Hung-Ming Wang

**Affiliations:** 1 Department of Medical Informatics, Tzu Chi University, 701, Sec. 3, Zhong Yang Rd., Hualien 970, Taiwan; 2 Institute of Medical Sciences, Tzu Chi University, Hualien 970, Taiwan; 101325107@gms.tcu.edu.tw; 3 Department of Chinese Medicine, Hualien Tzu Chi Hospital, Buddhist Tzu Chi Medical Foundation, Hualien 970, Taiwan; hsuanshushen@gmail.com; 4 Sports Medicine Center, Hualien Tzu Chi Hospital, Buddhist Tzu Chi Medical Foundation, Hualien 970, Taiwan; 5 School of Post-Baccalaureate Chinese Medicine, Tzu Chi University, Hualien 970, Taiwan; 6 Department of Biochemistry, Tzu Chi University, Hualien 970, Taiwan; pengsy@mail.tcu.edu.tw

**Keywords:** hyperthyroidism, heart rate dynamics, complexity, multiscale entropy analysis

## Abstract

Studying heart rate dynamics would help understand the effects caused by a hyperkinetic heart in patients with hyperthyroidism. By using a multiscale entropy (MSE) analysis of heart rate dynamics derived from one-channel electrocardiogram recording, we aimed to compare the system complexity of heart rate dynamics between hyperthyroid patients and control subjects. A decreased MSE complexity index (CI) computed from MSE analysis reflects reduced system complexity. Compared with the control subjects (*n* = 37), the hyperthyroid patients (*n* = 37) revealed a significant decrease (*p* < 0.001) in MSE CI (hyperthyroid patients 10.21 ± 0.37 versus control subjects 14.08 ± 0.21), sample entropy for each scale factor (from 1 to 9), and high frequency power (HF) as well as a significant increase (*p* < 0.001) in low frequency power (LF) in normalized units (LF%) and ratio of LF to HF (LF/HF). In conclusion, besides cardiac autonomic dysfunction, the system complexity of heart rate dynamics is reduced in hyperthyroidism. This finding implies that the adaptability of the heart rate regulating system is impaired in hyperthyroid patients. Additionally, it might explain the exercise intolerance experienced by hyperthyroid patients. In addition, hyperthyroid patients and control subjects could be distinguished by the MSE CI computed from MSE analysis of heart rate dynamics.

## 1. Introduction

Hyperthyroidism is a clinical syndrome resulting from excessive thyroid hormones in the body. Patients with hyperthyroidism present symptoms and signs induced by a hypermetabolic state due to the action of the excessive thyroid hormones. Palpitation and tachycardia are frequent cardiac manifestations in hyperthyroid patients. Studying heart rate dynamics would help understand the effects caused by a hyperkinetic heart in patients with hyperthyroidism. Previous studies mainly used linear analyses of heart rate variability (HRV) to investigate the heart rate dynamics in hyperthyroid patients [1,2,3]. However, human physiological regulating systems consist of multiple mutually interacting controlling loops and operate under a nonlinear mechanism. Using linear HRV analysis alone might not fully elucidate the characteristics of the heart rate dynamical system in hyperthyroidism.

A complex system consists of many components interacting with each other and their environment in multiple ways. A physiological system is composed of many mutually, nonlinearly interacting parts, and it has the adaptability to finely adjust itself to accommodate internal or external environmental changes. Biological physiological systems including the heart rate dynamical system have the characteristics of complex systems and could be regarded as complex systems [4]. In general, complex systems could be characterized by their system complexity.

Multiscale entropy (MSE) analysis has been utilized to quantify the complexity of complex systems [5]. The MSE complexity index (CI) computed from MSE analysis indicates the system complexity [6]. A decreased MSE CI reflects reduced system complexity. The analysis of MSE has been adopted to investigate the complexity of human physiological dynamical systems such as heart rate [5,7,8,9,10,11,12], electroencephalogram (EEG) [13,14,15,16,17,18], gait [19], posture [6,20,21], and others [22] in various kinds of patients. These studies have observed a common phenomenon, that reduced complexity for the studied systems is related to disease or aging.

Hyperthyroid patients have an impaired heart rate dynamical system, as evidenced by previous HRV studies [1,2,3]. Up to the present, there has not been a study in using MSE analysis to investigate the heart rate dynamical system in patients with hyperthyroidism. The study objective was to use MSE analysis to compare the system complexity of heart rate dynamics between the hyperthyroid patients and the control subjects. We hypothesized that the system complexity of heart rate dynamics is reduced in the hyperthyroid patients.

## 2. Methods

### 2.1. Subjects

From the outpatient clinic of a teaching hospital, we enrolled 37 newly diagnosed hyperthyroid Graves’ disease patients and 37 healthy normal control subjects. The hyperthyroid patients and the control subjects were matched for age (30 ± 1 versus 29 ± 1 years, hyperthyroid versus control, means ± SE), sex (4 males and 33 females versus 4 males and 33 females), and body mass index (20.8 ± 0.4 versus 22.0 ± 0.5 kg/m^2^). Graves’ disease was diagnosed based on clinical manifestations, thyroid function tests, immunological autoantibody tests, and thyroid scintigraphy with uptake data. We excluded individuals with cardiovascular disease, cardiac arrhythmia, pregnancy, and diabetes, and those with medication in use. The local institutional review board approved the study protocol, and informed consent was obtained from all participants. The study was performed in accordance with the principles in the Declaration of Helsinki.

### 2.2. Study Protocol and Procedures

All participants assured not taking alcoholic or caffeine-containing drinks for at least one day before the measurement. After 5 min rest, all participants received one-channel electrocardiography in lying for 30 min in a quiet room during daytime. Participants were regulated to breathe naturally and to fully relax, as well as not to speak and not to fall asleep during the process of electrocardiogram (ECG) recording. For the hyperthyroid patients, ECG recording was performed after diagnosis and before receiving any treatment. The analog ECG recorded from the electrocardiograph was immediately digitized by a 16-bit analog-to-digital converter using 500 Hz sampling frequency and was stored into a personal computer for further off-line analysis. The R waves were located first. Subsequently, premature beats and artifacts were deleted. The ECG recordings were excluded if the deletion percentage was >5%. The time intervals measured from the consecutive adjacent R waves were resampled at 4 Hz to obtain a sequence of normal R-R intervals for subsequent analysis.

### 2.3. Linear Analysis

From the normal R-R interval sequence, the time domain parameters of HRV: mean R-R interval, standard deviation of the R-R intervals (SDNN), and root mean square of successive differences between adjacent R-R intervals (RMSSD) as well as the frequency domain parameters of HRV: Total power (TP) (0–0.5 Hz), very low frequency power (VLF) (0–0.04 Hz), low frequency power (LF) (0.04–0.15 Hz), high frequency power (HF) (0.15–0.5 Hz), LF in normalized units (LF%), HF in normalized units (HF%), and the ratio of LF to HF (LF/HF) were computed by a self-developed software coded in MATLAB according to the HRV Task Force guideline [23].

### 2.4. Multiscale Entropy Analysis

In general, the MSE analysis of an ordered sequence (the normal R-R interval sequence for our study) includes three steps [5].

First, from a total *n* points sequence, $a_{k},\;k=1,\;2,\ldots ,n$, we constructed a set of consecutive coarse-grained sequences by the equation

$$x_{i}^{\left(\tau \right)}=\frac{1}{\tau }\sum_{k=(i-1)\tau +1}^{i\tau }a_{k},\;\;\;\tau =1,\;2,\ldots ,M;\;\;\;i=1,\;2,\ldots ,[\frac{n}{\tau }],$$

where *τ* is the scale factor, *M* is the given largest scale factor, and $\left[\frac{n}{\tau }]\;(=\mathrm{the}\;\mathrm{greatest}\;\mathrm{integer}\leq \frac{n}{\tau }\right)$ is the length of each coarse-grained sequence. Thus, from the original sequence, we obtained a total of *M* coarse-grained sequences.

Second, we calculated sample entropy for each coarse-grained sequence [24]. By given a template length *m* and a tolerance level *r*, sample entropy *S* is calculated as

$$S=-\ln\frac{U^{m+1}(r)}{U^{m}(r)},\;$$

where $U^{m}(r)$ is the probability that two *m* length sequences are similar within a tolerance level *r,* and $U^{m+1}(r)$ is the probability that two *m* + 1 length sequences are similar within a tolerance level *r*. Additionally, these probability does not count self-matches. Therefore, we would obtain the sample entropy for the corresponding scale factor.

Third, we calculated MSE CI [6] by summing sample entropy from *τ* = 1 to *τ* = *M* as

$$\mathrm{CI}=\sum_{\tau =1}^{M}S_{\tau },\;$$

where *S_τ_* is the sample entropy corresponding to the scale factor *τ*. For the calculation of sample entropy, we chose *m* = 2 and *r* = 0.15 of the standard deviation of the original sequence. In addition, the largest scale factor was set at *M* = 9 [24]. A self-developed software coded in MATLAB was used for MSE analysis.

### 2.5. Assays

In response to one of the anterior pituitary hormones, the thyroid-stimulating hormone (TSH), the thyroid gland secretes the thyroid hormones triiodothyronine (T3) and thyroxine (T4). In clinical practice, serum or plasma thyroid hormone measurement includes the free T3 (FT3) (and/or free T4 (FT4)) which is the T3 (and/or T4) not bound with proteins and the total T3 (T3) (and/or total T4 (T4)), which is the total amount of both bound and unbound T3 (and/or T4). Serum T3, T4, FT3, FT4 and TSH were quantified using a luminescent immunoassay (Vitros assay, Ortho-Clinical Diagnostics, UK).

### 2.6. Statistical Analysis

Comparisons of parameters for heart rate dynamics between the hyperthyroid patients and the control subjects were performed with the Mann–Whitney *U* test. Correlations were calculated by Spearman’s correlation coefficient. A *p*-value < 0.05 was considered significant. Data were expressed as means ± SE. The statistical software SPSS version 20.0 (IBM, Armonk, NY, USA) was used to analyze the data.

## 3. Results

High serum concentrations of thyroid hormones T3 (8.74 ± 0.45 nmol/L), (1.49–2.60, reference range); T4 (263.7 ± 9.1 nmol/L), (71.2–141); FT3 (30.43 ± 0.98 pmol/L), (4.26–8.10); FT4 (71.9 ± 2.7 pmol/L), (10.0–28.2); and a low serum concentration of TSH (0.006 ± 0.002 mIU/L), (0.465–4.68) were noted in the hyperthyroid patients.

**Figure 1:**
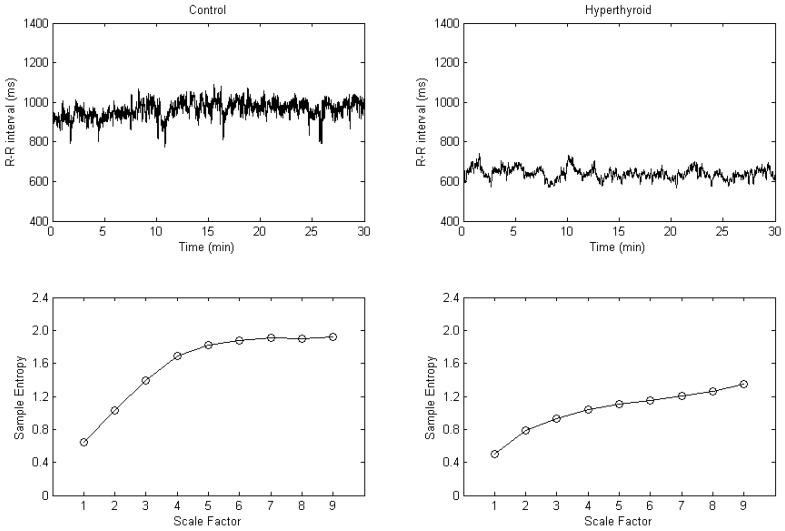
R-R interval tachograms (**upper**) and multiscale entropy plots (**lower**) in a control subject (**left**) and in a hyperthyroid patient (**right**).

The 30 min R-R interval tachograms and the MSE plots in a control subject and in a hyperthyroid patient are shown in Figure 1. The hyperthyroid patient compared with the control subject presented a grossly lesser fluctuation in the R-R interval tachogram (Figure 1). By plotting sample entropy to a scale factor from 1 to 9, the MSE plot was obtained. Corresponding to each scale factor in the MSE plots, the sample entropy was decreased in the hyperthyroid patient compared with the control subject (Figure 1).

The hyperthyroid patients exhibited significant differences (*p* < 0.001) as compared with the control subjects in the following heart rate dynamics parameters: decrease of mean R-R interval, SDNN, RMSSD, TP, VLF, LF, HF, and HF%; increase of LF% and LF/HF (Table 1). Moreover, the hyperthyroid patients compared with the control subjects revealed a lower MSE CI (hyperthyroid patients 10.21 ± 0.37 versus control subjects 14.08 ± 0.21, *p* < 0.001, Table 1).

**Figure 2:**
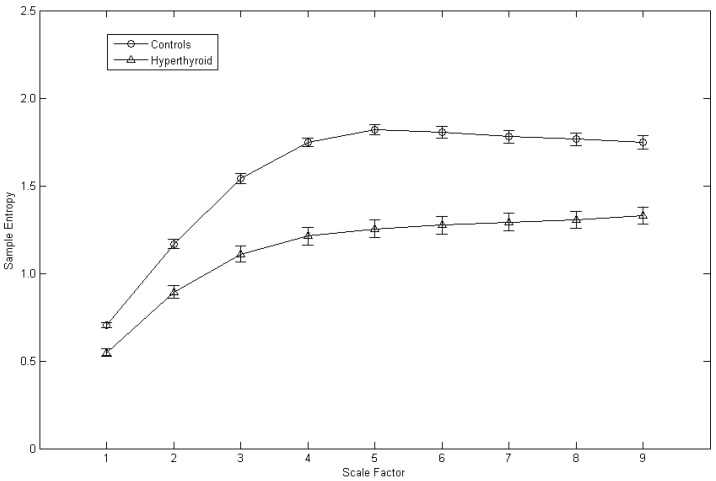
Average multiscale entropy plot of the control subjects (*n* = 37) and the hyperthyroid patients (*n* = 37). Symbols (circles for the control subjects and triangles for the hyperthyroid patients) represent the means of sample entropy with respect to scale factor in each group. Bars represent the standard error of the mean.

The average MSE plot of the control subjects and the hyperthyroid patients is shown in Figure 2. Corresponding to each scale factor, the mean of the sample entropy was calculated for all the control subjects and for all the hyperthyroid patients, respectively. The hyperthyroid patients (*n* = 37) compared with the control subjects (*n* = 37) exhibited lesser means of the sample entropy in all the scale factors from 1 to 9 (Figure 2 and Table 1).

For correlation analysis, MSE CI was negatively correlated with the serum concentrations of thyroid hormones T3 (*r* = −0.485, *p* = 0.002), T4 (*r* = −0.540, *p* = 0.001), FT3 (*r* = −0.382, *p* = 0.020), and FT4 (*r* = −0.576, *p* < 0.001) in the hyperthyroid patients.

## 4. Discussion

Our results showed a decreased MSE CI in the hyperthyroid patients compared with the control subjects via MSE analysis of the heart rate dynamical system. Such a finding suggests that the system complexity of heart rate dynamics is reduced in hyperthyroidism.

Hyperthyroidism presents symptoms and signs resembling those of a hyperadrenergic state. In addition, patients with hyperthyroidism manifest palpitation, tachycardia, and a higher prevalence of atrial fibrillation. The analysis of HRV offers a sensitive and non-invasive tool for the assessment of autonomic control of the heart [23,25,26]. In particular, the spectral analysis of HRV could correlate the respective effects of sympathetic and vagal control of the heart to different spectral powers, as indicated by the HRV spectrum [23,25]. Previous studies used the analysis of HRV to characterize the heart rate dynamics in the hyperthyroid patients. These studies mainly used a linear analysis of HRV and found that hyperthyroidism is characterized by both increased sympathetic and decreased vagal modulations of heart rate [2,3]. Our results of the linear HRV analysis are consistent with this finding, which might explain the apparent hyperadrenergic manifestations in patients with hyperthyroidism. However, similar to other physiological systems, the heart rate dynamical system is nonlinearly regulated by many mutually interacting systems such as neural, endocrine, etc. The method of nonlinear signal analysis would give new insight into the understanding of the heart rate dynamical system.

The heart rate dynamical system, a neuroautonomically regulated system, has the characteristics of complex systems and is regarded as a complex system [4]. In general, system complexity has been used to characterize complex systems. The complexity of a physiological complex system reflects its adaptability to the environment [4]. A healthy physiological system would have proper adaptability, similar to a robust complex system that could finely tune itself to perturbations. In response to internal or external changes to the system, a healthy physiological system could reactively adjust its states to adapt stressors and form a relatively stable homeostatic state. By contrast, a pathologic or aging system, due to the disruption of the regulating loop, would have impaired adaptability to stimuli and could not adequately respond to the changes. The system would not be able to maintain a homeostatic state and would reveal a reduced system complexity.

The MSE analysis, a nonlinear signal analysis, has been used to quantify the complexity of complex systems [5]. It computes the sample entropy [24] of a set of signals generated after manipulating the original time sequence signal with the coarse graining technique [5]. Afterwards, MSE CI is obtained by summing all sample entropy over several defined scale factors [6]. It denotes the system complexity of the original time sequence. A signal presenting lesser irregularity over multiple time scales would have decreased MSE CI and thus indicates reduced system complexity. For a physiological complex system, a reduced system complexity reflects the impaired adaptability of the system.

Using the analysis of MSE, a reduced system complexity of heart rate dynamics has been noted in patients with heart failure [11], diabetes [27], chronic kidney disease [28], major depression [29,30], stroke [31], as well as in aging [5], etc. Moreover, previous MSE analysis studies showed that reduced system complexity of heart rate dynamics could predict mortality in patients with trauma [32], congestive heart failure [33], and chronic kidney disease receiving dialysis [34,35]. In addition, MSE analysis of heart rate dynamics could predict stroke-in-evolution in acute ischemic stroke patients [10].

With MSE analysis, we are the first to show that hyperthyroid patients compared with control subjects exhibited a decreased MSE CI in their heart rate dynamics. The decreased MSE CI signifies the increased repetitions of heart rate fluctuating patterns in the heart rate sequence and therefore indicates a reduced system complexity of heart rate dynamics in patients with hyperthyroidism. Furthermore, this implies that the adaptability of the heart rate dynamical system is impaired in the hyperthyroid patients. Our findings are in agreement with previous MSE analysis studies and support the viewpoint that reduced complexity is related to disease and aging.

This reduced system complexity of heart rate dynamics could be attributed to the sustained stimulation of the excessive thyroid hormones on the heart in the hyperthyroid patients. This continuing stimulation leads to the impairment of the reactivity of the heart, and makes the hyperthyroid heart not able to respond adequately to additional external stimuli. Due to the impaired adaptability of the heart rate dynamical system, the hyperthyroid patients could not tolerate the exercise intensity that fits for normal subjects. Therefore, they exhibit exercise intolerance. In addition, our results showed that the MSE CI computed from the MSE analysis of the heart rate dynamical system could differentiate hyperthyroid patients from the control subjects. Future studies could be designed to justify whether MSE CI could be utilized as an indicator for monitoring therapeutic effects in the hyperthyroid patients.

For the correlation study, the MSE CI revealed significant negative correlations with the serum concentrations of all the thyroid hormones T3, T4, FT3, and FT4 in patients with hyperthyroidism. This discloses that MSE CI could indirectly signify the degree of the hyperthyroid state in the hyperthyroid patients.

There are several limitations to the present study. First, the case number is small in this study. This might interfere with the statistical power of the results. Second, we enrolled only hyperthyroid Graves’ disease patients and excluded other etiologies of the hyperthyroid patients. The results of this study should be inferred cautiously with other hyperthyroid patients.

In conclusion, besides cardiac autonomic dysfunction, decreased MSE CI was noted in the hyperthyroid patients compared with the control subjects. Such a finding suggests that the system complexity of heart rate dynamics is reduced in hyperthyroidism. Moreover, it implies that the adaptability of heart rate regulating system is impaired in hyperthyroid patients. Additionally, it might explain the exercise intolerance experienced by hyperthyroid patients. In addition, hyperthyroid patients and control subjects could be distinguished by the MSE CI computed from MSE analysis of heart rate dynamics.

## Figures and Tables

**Table 1:** Parameters of heart rate dynamics in control subjects and hyperthyroid patients.

	Controls	Hyperthyroid	*p*-Value
Mean RRI (ms)	865 ± 16	608 ± 14	<0.001
SDNN (ms)	54 ± 4	25 ± 2	<0.001
RMSSD (ms)	42 ± 4	9 ± 1	<0.001
TP (ms^2^)	3054 ± 488	772 ± 143	<0.001
VLF (ms^2^)	1566 ± 214	598 ± 112	<0.001
LF (ms^2^)	748 ± 146	133 ± 28	<0.001
HF (ms^2^)	740 ± 165	40 ± 9	<0.001
LF% (nu)	50.49 ± 1.67	77.31 ± 1.78	<0.001
HF% (nu)	49.51 ± 1.67	22.69 ± 1.78	<0.001
LF/HF	1.10 ± 0.07	4.63 ± 0.49	<0.001
CI	14.08 ± 0.21	10.21 ± 0.37	<0.001
S_1_	0.70 ± 0.01	0.55 ± 0.02	<0.001
S_2_	1.17 ± 0.03	0.89 ± 0.04	<0.001
S_3_	1.54 ± 0.03	1.11 ± 0.05	<0.001
S_4_	1.75 ± 0.03	1.21 ± 0.05	<0.001
S_5_	1.82 ± 0.03	1.25 ± 0.05	<0.001
S_6_	1.81 ± 0.03	1.27 ± 0.05	<0.001
S_7_	1.78 ± 0.04	1.29 ± 0.05	<0.001
S_8_	1.77 ± 0.04	1.31 ± 0.05	<0.001
S_9_	1.75 ± 0.04	1.33 ± 0.05	<0.001

Mean RRI = mean R-R interval; SDNN = standard deviation of the R-R intervals; RMSSD = root mean squares of the successive differences of the R-R intervals; TP = total power; VLF = very low frequency power; LF = low frequency power; HF = high frequency power; LF% = low frequency power in normalized units; HF% = high frequency power in normalized units; LF/HF = ratio of LF to HF; CI = multiscale entropy complexity index; S_1__–9_ = sample entropy calculated at scale factor 1–9 respectively. Data are means ± SE. Differences between means were assessed by Mann–Whitney *U* test.

## Data Availability

The data presented in this study are available on reasonable request from the corresponding author. The data are not publicly available due to ethics and privacy.
